# Enhancement of External Rotation after Latissimus Dorsi Tendon Transfer (LDTT): A Cadaveric Study

**DOI:** 10.3390/medicina57040305

**Published:** 2021-03-24

**Authors:** José M. Silberberg, Alessandro Nilo, Jorge Roces-García

**Affiliations:** 1 Orthopaedic Surgery and Traumatology Head Department, Sports Medicine Unit, Clínica Universidad de Navarra, 28027 Madrid, Spain; 2 Orthopaedic Surgery and Traumatology Department, Chief of Upper Limb Unit, Hospital General Regional N1, 97155 Mérida, Mexico; a.nilofulvi@gmail.com; 3 Department of Construction and Manufacturing Engineering, Polytechnic School of Engineering of Gijón, University of Oviedo, Pedro Puig Adam s/n, ED06, 33203 Gijón, Spain; rocesjorge@uniovi.es

**Keywords:** cadaver, external rotation, latissimus dorsi tendon transfer (LDTT), rotator cuff, shoulder

## Abstract

*Background and objectives*: Massive rotator cuff tears compromise shoulder mobility function and cannot be directly repaired. Latissimus dorsi tendon transfer (LDTT) is a therapeutic alternative suitable for the treatment of rotator cuff tears that helps to restore external shoulder rotation. Cadaver models have been used for studying the effects of LDTT and procedural variations, but, to the best of our knowledge, none of them have been validated. The aim of our study was to validate a novel cadaver model while verifying the effects of LDTT on external rotation. *Materials and Methods*: Two groups were included in the study: a cadaver group and a control group made up of healthy volunteers, which were used for the validation of the cadaver model. Baseline external rotation measurements were performed with both groups, after which a massive rotator cuff tear was inflicted and repaired with LDTT in the cadaver group. Their postoperative external rotation was evaluated using three different tests. *Results*: No statistically significant differences were found between the baseline measurements of the two groups, and postoperative external rotation was significantly higher after LDTT in all cases but one. *Conclusions:* Cadaver models were validated, since they had a similar preoperative external rotation to healthy volunteers. Moreover, they allowed us to demonstrate the effect of LDTT on external shoulder rotation.

## 1. Introduction

The rotator cuff not only contributes to the motion of the shoulder joint (abduction, internal rotation, and external rotation), but also to glenohumeral stability, preventing the proximal migration of the humeral head [1,2]. When a rotator cuff injury occurs, shoulder motion may be compromised, particularly in the event of a posterosuperior lesion, i.e., a lesion affecting the supraspinatus and infraspinatus tendons, resulting in a loss of external rotation. Massive tears (which involve two or more tendons or are larger than 5 cm) are often not amenable to direct repair, especially when associated with muscle atrophy and fatty infiltration. In these cases, a latissimus dorsi tendon transfer (LDTT) has emerged as a promising therapeutic alternative [3,4,5,6,7,8,9,10].

The latissimus dorsi is a vast muscle that attaches to the distal portion of the medial bicipital groove between the pectoralis major and subscapularis tendons. Its normal function includes internal rotation, adduction, and backward extension of the humerus [11,12]. In LDTT, the latissimus dorsi tendon is harvested from its medial humeral insertion and reattached to the supraspinatus footprint. This allows the latissimus dorsi to restore external shoulder rotation and prevent the superior migration of the humeral head, thus improving deltoid function [3,5,7,12,13].

Although cadaver models cannot completely match a live patient, they can be useful for developing improvements in surgical techniques or training surgeons. Previous studies have used cadaver models for the assessment of LDTT since they are a rapid and direct way to visualize the effects of procedural variations on shoulder functionality. However, to the best of our knowledge, none of these models have been validated [14,15,16,17,18].

For this reason, the present study aimed to validate a novel cadaver model by comparing it to healthy living subjects, while also ascertaining that LDTT does restore external rotation after a massive rotator cuff tear.

## 2. Materials and Methods

### 2.1. Study Groups

Our study was conducted with two groups. The cadaver group was made up of three fresh frozen cadavers with a mean age of 64.3 years (range, 62–67 years). They were stored at −20 °C before testing and thawed for 12 h at room temperature. The clinical practice complies with the ethical and legal requirements for testing medical devices on human cadavers, as well as the medical tests necessary to ensure the safety of all participants. The anatomical material used comes from local donors, provided by the Catholic University of Valencia. When selecting the cadavers, specimens with previous conditions involving the glenohumeral and/or scapulothoracic joints, limited range of motion, rotator cuff tears, or previous shoulder surgery were excluded. All three cadavers were upper hemibody specimens with an eviscerated ribcage, full head and neck, and all the static and dynamic stabilizers of the glenohumeral and scapulothoracic joints of both extremities were present, including the skin.

The control group, made up of 50 healthy volunteers, was used to validate the cadaver models. To this effect, a representative sample of 50 individuals was selected following the selection criteria shown in Table 1.

Out of the 50 healthy volunteers, 23 (46%) were male and 27 (54%) were female. Their baseline characteristics are shown in Table 2.

### 2.2. Measurement Device

**Figure 1:**
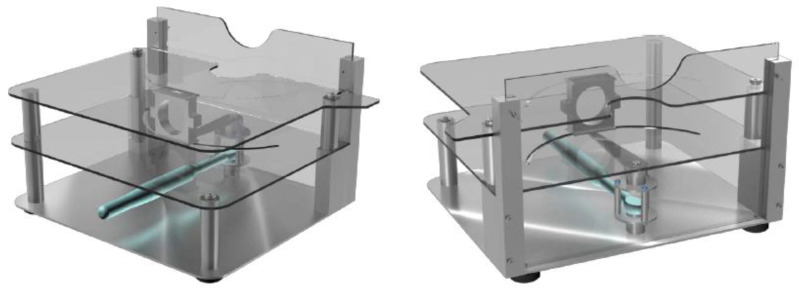
Electromechanical measurement device.

All measurements were made with an electromechanical device (shown in Figure 1) that allowed us to easily record the external torque applied to the upper extremity and the resulting external rotation.

To ensure the accuracy of the measurements, all subjects were placed next to the device with their axilla resting on its height-adjustable lateral panel. Their arms were placed on the rotation axis of the device, preventing any contact between the elbow and the equipment to minimize friction. The subject’s wrists were attached to a clamp located on the end of the rotating rod, which mimicked the external rotation movement of the shoulder. To ensure uniformity between measurements, the torque exerted on the subject’s arm was applied through a programmable digital torque wrench. All measurements were made with the subject’s arm in neutral rotation, held close to the chest.

**Figure 2:**
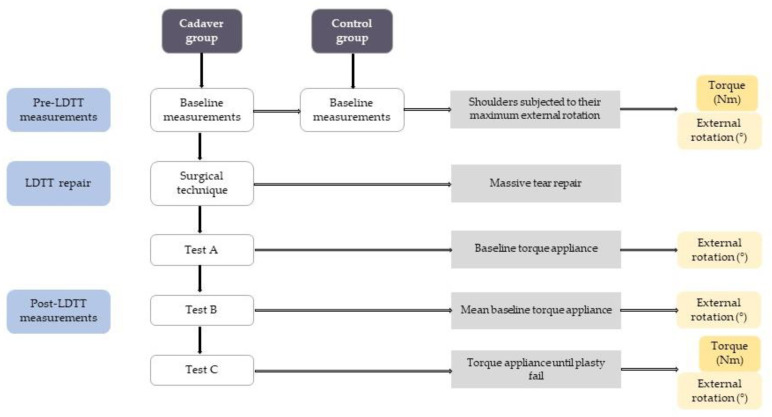
Methodological flowchart. Purple squares indicate study groups, blue squares show the timeline, and white squares represent each main step of the study. Connected by an arrow to each step are the brief explanation (grey) and the variables being measured (yellow). LDTT, latissimus dorsi tendon transfer.

All steps of the study are detailed in the following subsections and summarized in Figure 2.

### 2.3. Pre-LDTT Measurements

**Figure 3:**
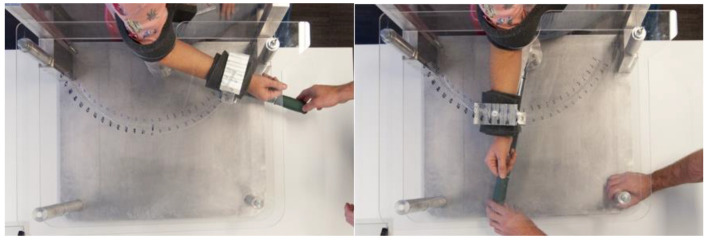
Measurement of the external rotation of one of the subjects in the control group using the measurement device.

Both groups were subjected to baseline measurements, which involved forcing the maximum external rotation of both arms for each subject until resistance was felt in the cadaver group or verbally expressed in the control group (Figure 3).

### 2.4. Surgical Technique

A massive posterolateral tear was created to both cadaveric shoulders in each specimen, which were subsequently repaired using a LDTT procedure. Subjects were placed laterally, and the greater tuberosity was debrided to induce bleeding in the latissimus dorsi recipient site. A longitudinal incision was made over the posterior axillary pillar, separating the latissimus dorsi tendon from its humeral insertion site. The tendon was retrogradely resected from the thoracic wall, sparing the neurovascular pedicle. Non-resorbable sutures were applied on each side of the tendon. Mobilization of the tendon was deemed to be appropriate when the tip of the tendon surpassed the posterolateral border of the acromion by 2 cm. The latissimus dorsi tendon was transported to the subacromial space through the space between the infraspinatus, the teres minor, and the posterior deltoid. The two sutures (medial and lateral) on the side of the latissimus dorsi graft were anchored to the greater tuberosity with knotless anchors.

### 2.5. Post-LDTT Measurements

To restore the normal viscoelastic properties of soft tissues, five maximum internal and external rotation movements were performed on each shoulder. All movements were carried out by applying the minimum torque needed for shoulder movement (2.2 N·m) that we estimated using free validated simulation software (OpenSim [19,20]). This allowed us to restore physiological properties without compromising the anatomical structures.

Three tests were then conducted with the cadaver group to evaluate postoperative external rotation:

− Test A: Shoulders were subjected to the same torque values applied at baseline.
− Test B: Shoulders were subjected to the mean torque value applied at baseline.
− Test C: Sustained torque was applied until the repaired tendon avulsed from its insertion on the greater tuberosity. This was used to determine the maximum torque that the reconstruction could endure.

### 2.6. Statistical Analysis

Data are presented as mean and standard error of the mean (SEM). An independent samples *t*-test was used for the comparative analysis of the baseline measurements between both groups, while a paired-samples *t*-test was used for the comparison of baseline measurements with Tests A and B in the cadaver group. These differences are represented with scatterplots and kernel density plots. Kernel density plots show the continuous distribution of a single variable as a flattened histogram.

All statistical analyses were carried out using the Stata 15 software package (StataCorp, College Station, TX, USA), establishing 80% power (1-β) for all tests. A *p*-value < 0.05 was considered statistically significant in all cases.

## 3. Results

The baseline measurements for both study groups were compared to identify differences in their preoperative performance and to determine the validity of the cadaver models. The baseline mean torque was 18.9 N·m (SEM = 0.86) in the control group and 21.4 N·m (SEM = 1.35) in the cadaver group, while the mean external rotation was 99.8° (SEM = 1.18) in the control group and 103.2° (SEM = 2.38) in the cadaver group. No statistically significant differences were found between the two groups for the maximum torque (*p* = 0.23) or the external rotation (*p* = 0.25).

**Figure 4:**
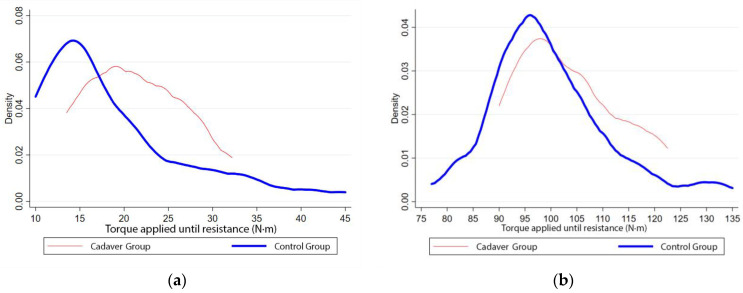
Continuous distribution comparing baseline measurements of the control (blue) and cadaver (red) groups in terms of the torque required to achieve maximum external rotation (**a**) and said external rotation (**b**).

Figure 4a shows a continuous distribution representation of the torque required to achieve maximum preoperative external rotation in both groups, and Figure 4b shows said maximum external rotation for both groups. All curves show a similar pattern, with a slight rightward shift in the cadaver group, probably due to muscle distention, which is typically observed in cadaveric specimens.

**Figure 5:**
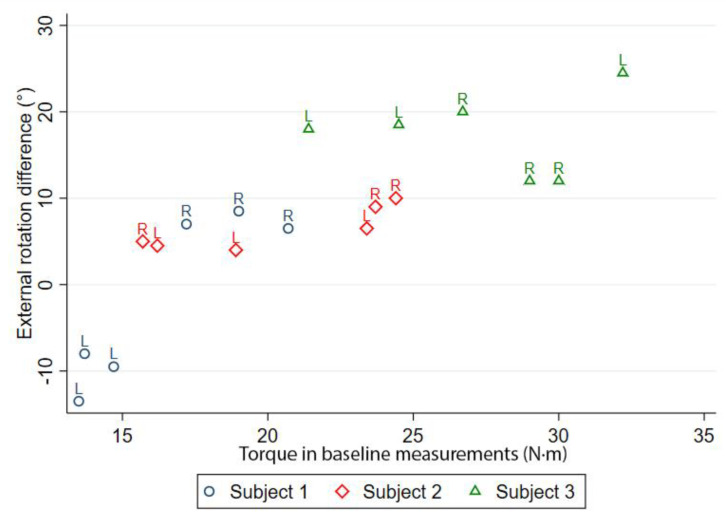
Differences in external rotation between baseline and post-LDTT (Test A) in cadaveric specimens for the maximum torque values. The values for each arm are shown separately for each of the three subjects (L = left; R = right). Three measurements per arm were recorded for both arms of the cadaveric specimens.

Figure 5 shows the absolute differences in external rotation before and after LDTT for the cadaver group, as obtained in Test A. In all cases, except for the left shoulder of subject 1, external rotation increased after the procedure. There were statistically significant differences between the external rotation achieved pre- and post-operatively (*p* < 0.001), with a mean increase in external shoulder rotation of 7.5° (SEM = 3.24).

**Figure 6:**
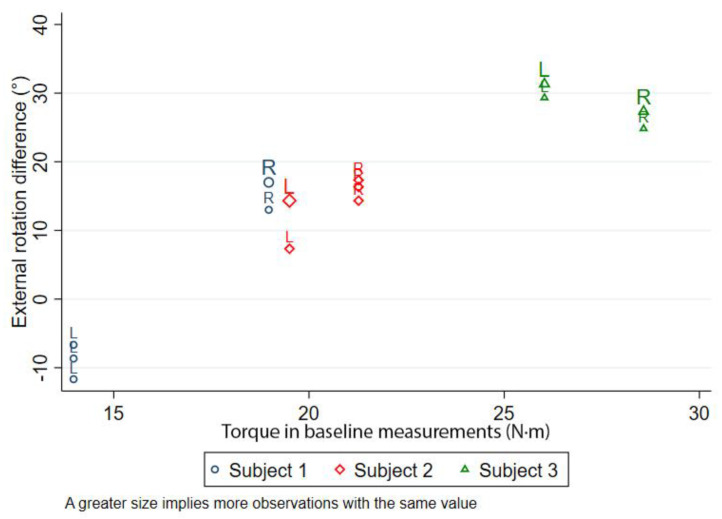
Differences in external rotation between baseline and post-LDTT performance (Test B) in the cadaveric specimens for maximum mean preoperative external rotation values. The values for each arm are shown separately for each of the three subjects (L = left; R = right). Three measurements per arm were recorded for both arms of the cadaveric specimens.

The differences between the external rotation before and after LDTT as measured in Test B are shown in Figure 6. Again, except for the left shoulder of subject 1, post-LDTT external rotation increased in all cases. The cadaveric specimens achieved a mean increase of 15.3° (SEM = 3.26) in the external rotation of the shoulder, which was considered to be a significant increase compared to their baseline measurement (*p* = 0.03).

We performed Test C to determine the maximum torque that the reconstruction could withstand before breaking. However, only three out of the six tendons avulsed from their insertion during Test C, despite the mean torque being applied (138 N·m; SEM = 6.01), and the subsequent external rotations (33.0°; SEM = 2.19) were significantly higher than those applied in Tests A and B.

## 4. Discussion

When using cadaver models, the specimens have to represent close to physiologic anatomy behavior. Model validation is therefore very specific to an application and implies that selection criteria appropriate for the use of the models were used [18].

The presented cadaveric specimens appear to be a valid model for the study of external shoulder rotation, as no significant differences were found between the cadaver and control groups in baseline measurements. This is the first time that this comparison has been made in shoulders undergoing LDTT [11,15,17,21]. The values were slightly higher in the cadaver model, which permits a wider range of muscle movements, and have absence of pain.

Numerous alternatives have been reported for the treatment of rotator cuff tears, such as arthroscopic subacromial debridement, partial tendon repair, acromioplasty, allograft reconstruction, and muscle transfer. The choice of one technique over another mainly depends on the patient’s age and functional requirements, with LDTT representing a valid alternative for young active patients with high functional demands [6,10,13,22,23,24,25]. A wide number of studies confirm its efficacy, since it restores active external rotation of the shoulder, relieves pain, and improves shoulder torque and function [3,5,7,10,13,23,26].

External rotation was not only restored, but increased after LDTT, which was statistically significant when comparing baseline and postoperative measurements (Tests A and B). Active external rotation increase after LDTT has already been reported by several studies, but it should be noted that we exclusively measured passive external rotation, since we would need to apply an external active muscle contraction to measure active external rotation in cadaveric specimens. LDTT has a direct effect on muscle contraction (restoring active external rotation) and a tenodesis effect, descending the humeral head and improving glenohumeral kinematics, which could explain its effects on passive external rotation [10].

However, one of the six shoulders experienced a decrease in postoperative external rotation. This could be attributed to either a failure when performing the surgical technique or a non-diagnosed lesion, such as fatty infiltration in the teres minor or instability in the subscapularis tendon, which are known contraindications for LDTT [12,13,17,25,27]. Nevertheless, none of these indicators were stated in the specimens’ records, and we believe that this result does not overshadow the overall uniformity of the data. Moreover, only half of the reconstruction detached from their insertion point when subjected to significantly high torque, which proves their strength and stability.

The quantification of the range of motion of the shoulder is usually carried out using goniometers, although they require a certain skill by the clinician, and some authors believe that inter-observer and inter-instrument variability should not be disregarded [28,29,30]. Our measurement device constitutes an innovative alternative that is both comfortable for the patient and easy to interpret by the clinician. Based on the principles of goniometry, the device makes it possible to simultaneously measure two different parameters: applied torque and external shoulder rotation.

Overall, we present an original study validating cadaver models with intact shoulder joint structures that allowed us to verify the effects of LDTT on external shoulder rotation. We consider our cadaver model’s anatomical features and preservation method to be suitable for shoulder studies.

The main weakness of this study is a small number of cadaver models, which limits the power of our study. Although we believe that our results are remarkably uniform, they should be taken with caution. Future studies should focus on using the same cadaver models for studying LDTT variations or other shoulder surgeries.

## 5. Conclusions

Our cadaver models that were selected for studying external rotation of the shoulder after LDTT were validated, as no statistical differences were found when comparing their preoperative measurements with those of healthy volunteers. Moreover, they allowed us to verify that external rotation is not only restored, but increased after LDTT.

## Figures and Tables

**Table 1:** Inclusion and exclusion criteria for the control group.

**Inclusion Criteria**
• Aged between 18 and 65 years
• Signed informed consent
**Exclusion Criteria**
• Any diagnosed shoulder pathology that implied an unnatural limited range of motion, including glenohumeral and/or scapulothoracic joint condition, rotator cuff tears, or any previous shoulder surgery

**Table 2:** Baseline characteristics of the control group. Data are presented as mean (range).

	Male	Female
Age (years)	38.3 (25.0–61.0)	33.9 (21.0–44.0)
Weight (kg)	81.8 (62.0–98.0)	60.2 (47.0–78.0)
Height (cm)	177.7 (170.0–191.0)	165.1 (150.0–178.0)
BMI (Body Mass Index)	25.9 (21.2–30.8)	22.1 (17.7–29.7)

## Data Availability

The data presented in this study are available on request from the corresponding author. The data are not publicly available due to ethical restrictions.

## References

[1] Yamamoto N., Itoi E. (2015). A review of biomechanics of the shoulder and biomechanical concepts of rotator cuff repair. Asia-Pac. J. Sport Med. Arthrosc. Rehabil. Technol..

[2] Maruvada S., Madrazo-Ibarra A., Varacallo M. (2018). Anatomy, Rotator Cuff. StatPearls Publishing LLC. https://www.ncbi.nlm.nih.gov/books/NBK441844/.

[3] Grimberg J., Kany J. (2014). Latissimus dorsi tendon transfer for irreparable postero-superior cuff tears: Current concepts, indications, and recent advances. Curr. Rev. Musculoskelet. Med..

[4] Nové-Josserand L., Costa P.A., Liotard J.-P., Safar J.-F., Walch G., Zilber S. (2009). Results of latissimus dorsi tendon transfer for irreparable cuff tears. Orthop. Traumatol. Surg. Res..

[5] Osti L., Buda M., Andreotti M., Gerace E., Osti R., Massari L., Maffulli N. (2018). Arthroscopic-assisted latissimus dorsi transfer for massive rotator cuff tear: A systematic review. Br. Med. Bull..

[6] Oh J.H., Tilan J., Chen Y.-J., Chung K.C., McGarry M.H., Lee T.Q. (2012). Biomechanical effect of latissimus dorsi tendon transfer for irreparable massive cuff tear. J. Shoulder Elb. Surg..

[7] Donaldson J., Noorani A., Falworth M., Pandit A., Douglas T., Lambert S. (2011). Latissimus dorsi tendon transfers for rotator cuff deficiency. Int. J. Shoulder Surg..

[8] Wagner E.R., Woodmass J.M., Welp K.M., Chang M.J., Elhassan B.T., Higgins L.D., Warner J.J. (2018). Novel Arthroscopic Tendon Transfers for Posterosuperior Rotator Cuff Tears. JBJS Essent. Surg. Tech..

[9] Irlenbusch U., Bernsdorf M., Born S., Gansen H.-K., Lorenz U. (2008). Electromyographic analysis of muscle function after latissimus dorsi tendon transfer. J. Shoulder Elb. Surg..

[10] Zaidenberg C.R., Zaidenberg E.E., Pastrana M.J., Francisco F. (2017). Transferencia de dorsal ancho para el tratamiento de las lesiones masivas e irreparables del manguito rotador. [Latissimus dorsi transfer for treatment of massive and irreparable rotator cuff injuries.]. Rev. Asoc. Argent. Ortop. Traumatol..

[11] Ranade A.V., Rai R., Rai A.R., Dass P.M., Pai M.M., Vadgaonkar R. (2018). Variants of latissimus dorsi with a perspective on tendon transfer surgery: An anatomic study. J. Shoulder Elb. Surg..

[12] Clark N.J., Elhassan B.T. (2018). The Role of Tendon Transfers for Irreparable Rotator Cuff Tears. Curr. Rev. Musculoskelet. Med..

[13] Axe J.M. (2016). Tendon transfers for irreparable rotator cuff tears: An update. EFORT Open Rev..

[14] Henry P.D.G., Dwyer T., McKee M.D., Schemitsch E.H. (2013). Latissimus dorsi tendon transfer for irreparable tears of the rotator cuff. Bone Jt. J..

[15] Bargoin K., Boissard M., Kany J., Grimberg J. (2016). Influence of fixation point of latissimus dorsi tendon transfer for irreparable rotator cuff tear on glenohumeral external rotation: A cadaver study. Orthop. Traumatol. Surg. Res..

[16] Favre P., Loeb M.D., Helmy N., Gerber C. (2008). Latissimus dorsi transfer to restore external rotation with reverse shoulder arthroplasty: A biomechanical study. J. Shoulder Elb. Surg..

[17] Werner C.M., Zingg P.O., Lie D., Jacob H.A., Gerber C. (2006). The biomechanical role of the subscapularis in latissimus dorsi transfer for the treatment of irreparable rotator cuff tears. J. Shoulder Elb. Surg..

[18] Eisma R., Wilkinson T. (2014). From “Silent Teachers” to Models. PLoS Biol..

[19] Baillargeon E.M., Ludvig D., Sohn M.H., Nicolozakes C.P., Seitz A.L., Perreault E.J. (2019). Experimentally quantifying the feasible torque space of the human shoulder. J. Electromyogr. Kinesiol..

[20] Blache Y., Begon M. (2018). Influence of Shoulder Kinematic Estimate on Joint and Muscle Mechanics Predicted by Musculoskeletal Model. IEEE Trans. Biomed. Eng..

[21] Ackland D.C., Pandy M.G. (2011). Moment arms of the shoulder muscles during axial rotation. J. Orthop. Res..

[22] De Casas R., Lois M., Cidoncha M., Valadron M. (2014). Clinic and electromyographic results of latissimus dorsi transfer for irreparable posterosuperior rotator cuff tears. J. Orthop. Surg. Res..

[23] Gerber C., Gerardo M., Espinosa N. (2006). Latissimus dorsi transfer for the treatment of irreparable rotator cuff tears. JBJS.

[24] Ortmaier R., Hitzl W., Matis N., Mattiassich G., Hochreiter J., Resch H. (2017). Reverse shoulder arthroplasty combined with latissimus dorsi transfer: A systemic review. Orthop. Traumatol. Surg. Res..

[25] Cutbush K., Peter N.A., Hirpara K. (2016). All-Arthroscopic Latissimus Dorsi Transfer. Arthrosc. Tech..

[26] Henseler J.F., Nagels J., Nelissen R.G., De Groot J.H. (2014). Does the latissimus dorsi tendon transfer for massive rotator cuff tears remain active postoperatively and restore active external rotation?. J. Shoulder Elb. Surg..

[27] Gerhardt C., Lehmann L., Lichtenberg S., Magosch P., Habermeyer P. (2009). Modified L’Episcopo Tendon Transfers for Irreparable Rotator Cuff Tears: 5-year Followup. Clin. Orthop. Relat. Res..

[28] Kolber M.J., Mdt C. (2012). Original Research the Reliability and Concurrent Validity of Shoulder Mobility Measurements Using a Digital Inclinometer and Goniometer. Int. J. Sports Phys. Ther..

[29] Lee S.H., Yoon C., Chung S.G., Kim H.C., Kwak Y., Park H.-W., Kim K. (2015). Measurement of shoulder range of motion in patients with adhesive capsulitis using a Kinect. PLoS ONE.

[30] Cools A.M., De Wilde L., Van Tongel A., Ceyssens C., Ryckewaert R., Cambier D.C. (2014). Measuring shoulder external and internal rotation strength and range of motion: Comprehensive intra-rater and inter-rater reliability study of several testing protocols. J. Shoulder Elb. Surg..

